# Bilateral Dome-Shaped Macula with Serous Macular Detachment in a Child

**DOI:** 10.1155/2015/213968

**Published:** 2015-06-01

**Authors:** Zafer Cebeci, Nur Kir

**Affiliations:** Department of Ophthalmology, Istanbul Faculty of Medicine, Istanbul University, Capa, 34390 Istanbul, Turkey

## Abstract

Dome-shaped macula is a structural disorder and optical coherence tomography (OCT) helps us to confirm macular convexity. We describe the first case of bilateral dome-shaped macula in an 8-year-old boy with subretinal fluid. The patient was diagnosed using spectral-domain OCT and received indocyanine green angiography-guided half-fluence photodynamic therapy as treatment.

## 1. Introduction

Structural change of the macula in highly myopic eyes was first defined by Gaucher et al. using optical coherence tomography (OCT) in 2008 and was called “dome-shaped macula” [1]. In this condition, the normal shape of the macula detected on OCT is altered and inward bulging occurs in a dome-shaped formation.

Choroidal neovascularization, serous retinal detachment, and retinal pigment epithelial (RPE) detachments are the most common macular complications that can occur during follow-up of dome-shaped macula [1–4].

In this paper, we present the case of a child with dome-shaped macula in both eyes, complicated by serous detachment in one eye.

## 2. Case Report

**Figure 1:**
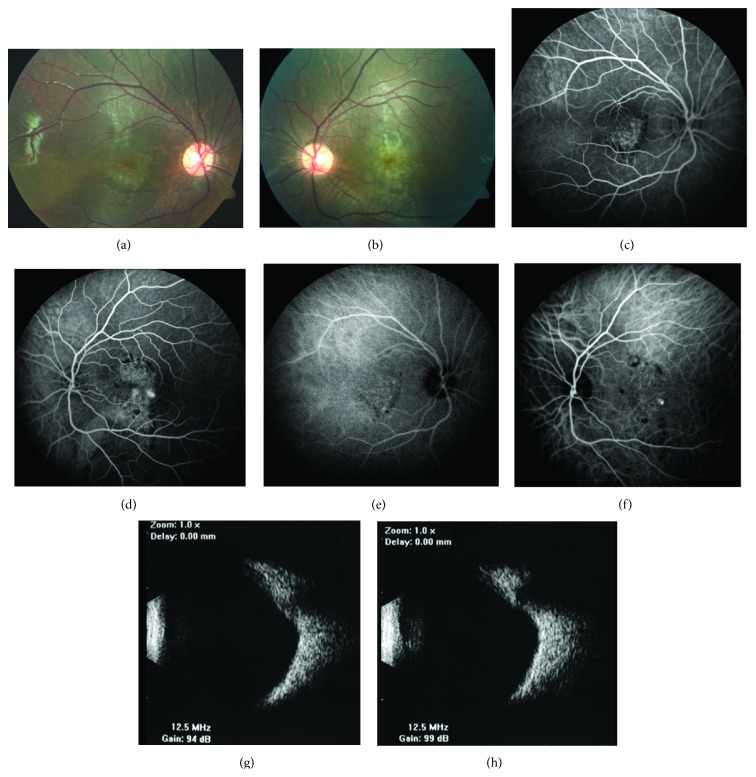
Color fundus photography showed retinal pigment epithelial (RPE) changes on macula in the right (a) and left (b) eye. Fluorescein angiography (FA) illustrates hyperfluorescence due to window defect in both eyes and hyperfluorescent foci on the left eye (c, d). Indocyanine green angiography (ICG-A) demonstrates mild hyperfluorescence due to RPE atrophy in both eyes and marked hyperfluorescent spot in the left eye (e, f). Ultrasonography showed no choroidal mass in both eyes (g, h).

**Figure 2:**
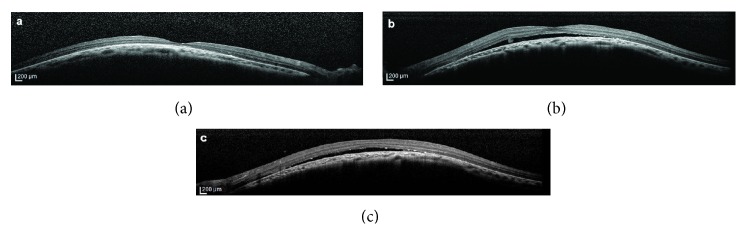
Spectral-domain optical coherence tomography showed dome-shaped macula in both eyes and serous retinal detachment with subretinal hyperreflective material on the nasal side of the left eye (a, b). OCT scan through the hyperfluorescent foci in the left eye showed subretinal fluid and RPE alterations (c).

**Figure 3:**
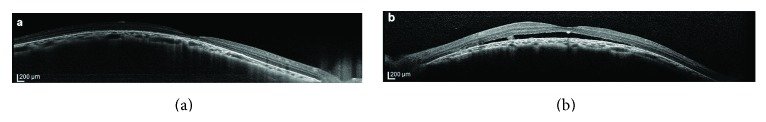
SD-OCT demonstrated dome-shaped macula in the right (a) and left eye (b) with persistence of serous retinal detachment in the left eye at the last follow-up.

An 8-year-old boy presented to our clinic with the complaint of decreased vision in the left eye. His ophthalmological and medical history were unremarkable. Best corrected visual acuities were 20/25 in the right eye and 20/32 in the left eye. Spheric equivalents were −4.50 in the right eye and −4.00 in the left eye. The anterior segment was normal bilaterally. Fundoscopy showed retinal pigment epithelial changes in the macula of both eyes (Figures 1(a) and 1(b)). Fluorescein angiography (FA) and indocyanine green angiography (ICG-A) (Spectralis, Heidelberg Engineering, Heidelberg, Germany) demonstrated hyperfluorescence due to window defect and surrounding irregular hypofluorescent areas in both macula, as well as hyperfluorescent foci temporal to the macula in the left eye (Figures 1(c), 1(d), 1(e), and 1(f)). Choroidal mass was ruled out with ultrasonography (Figures 1(g) and 1(h)). SD-OCT (Spectralis, Heidelberg Engineering, Heidelberg, Germany) revealed bilateral dome-shaped macula and serous detachment with a hyperreflective lesion nasally in the left eye (Figures 2(a), 2(b), and 2(c)). Subfoveal choroidal thickness was measured with EDI-OCT (Spectralis, Heidelberg Engineering, Heidelberg, Germany) and found 204 *μ*m and 158 *μ*m for right and left eye, respectively. With these findings, the patient was diagnosed with dome-shaped macula and subretinal fluid in the left eye. Half-fluence photodynamic therapy (PDT) was applied to the hyperfluorescent areas under the guidance of ICG-A. Over the one-year follow-up, his visual acuities remained the same and the subretinal fluid persisted. It had not resolved on SD-OCT at the last follow-up (Figures 3(a) and 3(b)).

## 3. Discussion

Dome-shaped macula is an obvious change in the configuration of the macula anteriorly that can be detected with OCT [1]. Fundoscopic examination cannot effectively identify a dome-shaped contour, and the diagnosis can be dismissed if OCT is not used.

Visual loss usually results from macular complications such as choroidal neovascularization and serous retinal detachment [1–4]. There may be diagnostic confusion with central serous chorioretinopathy (CSC) when subretinal fluid is detected. A fundoscopic appearance of RPE changes and FA and ICG-A images with a hyperfluorescent leakage point can simulate chronic CSC [1]. In a study of 48 eyes, subfoveal fluid was detected in 25 patients, and macular bulge was statistically increased and vision was decreased in this group of patients [2]. Serous detachment complication rates vary in the literature. Gaucher et al., Imamura et al., and Ohsugi et al. reported that 66.7%, 9%, and 10.2%, respectively, of the patients in their studies had subretinal fluid [1, 3, 4]. Abnormal bending of the macula with increased choroidal thickening and scleral thickening causing choroidal fluid outflow failure were the mechanisms blamed for the formation of subretinal fluid [1–3]. Fundoscopic and angiographic imaging modalities caused us to think our case was similar, with chronic CSC, but OCT confirmed the diagnosis of dome-shaped macula.

Treatment of serous retinal detachment in dome-shaped macula is not clearly defined because treated patients are only reported as case reports. Treatment options include photodynamic therapy, focal laser photocoagulation, and spironolactone [1, 5]. Chinskey and Johnson reported 2 dome-shaped maculas with subretinal fluid that were treated with half-fluence PDT [5]. Fluid was resolved with one session in one patient, and the other patient received PDT twice, showing a partial response after the second procedure. Dirani et al. treated two dome-shaped macula patients, who had complications with serous foveal detachment, with a mineralocorticoid receptor antagonist, spironolactone [6]. After effective treatment, they stated that a mineralocorticoid pathway may play a role in the formation of serous detachment in dome-shaped macula as seen in CSC. Spontaneous resolution of fluid is also reported in the literature [1]. We performed half-fluence PDT on our patient but did not obtain a satisfactory response, and the fluid did not resolve during the follow-up period. Further investigations on the treatment of serous macular detachment must be done in larger groups of patients with dome-shaped macula.

Most of the patients reported on have been over 20 years of age. The youngest patient with a dome-shaped macula was reported by Errera et al. [7]. A 12-year-old male with congenital stationary night-blindness was described in their study, and no complication was found.

In conclusion, our case is the youngest patient with dome-shaped macula reported in the literature, and this case was complicated by subretinal fluid. No proven treatment option exists for this macular configuration with serous retinal detachment, and we were unsuccessful using PDT to treat this complication. However, patients must be closely followed for any vision-threatening complications.

## References

[1] Gaucher D., Erginay A., Lecleire-Collet A. (2008). Dome-shaped macula in eyes with myopic posterior staphyloma. *The American Journal of Ophthalmology*.

[2] Caillaux V., Gaucher D., Gualino V., Massin P., Tadayoni R., Gaudric A. (2013). Morphologic characterization of dome-shaped macula in myopic eyes with serous macular detachment. *The American Journal of Ophthalmology*.

[3] Imamura Y., Iida T., Maruko I., Zweifel S. A., Spaide R. F. (2011). Enhanced depth imaging optical coherence tomography of the sclera in dome-shaped macula. *American Journal of Ophthalmology*.

[4] Ohsugi H., Ikuno Y., Oshima K., Yamauchi T., Tabuchi H. (2014). Morphologic characteristics of macular complications of a dome-shaped macula determined by swept-source optical coherence tomography. *American Journal of Ophthalmology*.

[5] Chinskey N. D., Johnson M. W. (2013). Treatment of subretinal fluid associated with dome-shaped macula. *Ophthalmic Surgery, Lasers & Imaging Retina*.

[6] Dirani A., Matet A., Beydoun T., Mantel I., Behar-Cohen F. (2014). Resolution of foveal detachment in dome-shaped macula after treatment by spironolactone: report of two cases and mini-review of the literature. *Clinical Ophthalmology*.

[7] Errera M.-H., Michaelides M., Keane P. A. (2014). The extended clinical phenotype of dome-shaped macula. *Graefe's Archive for Clinical and Experimental Ophthalmology*.

